# Systematic SARS-CoV-2-testing for asymptomatic cancer patients treated at a public healthcare tertiary centre in Brazil

**DOI:** 10.3332/ecancer.2021.1269

**Published:** 2021-07-26

**Authors:** Aline F Fares, Luiza A Fadul, Barbara Benetton, Mauricio L Nogueira, Marcia Lanza, Daniel V Araújo

**Affiliations:** 1 Department of Medical Oncology, Hospital de Base–HB Onco, Faculty of Medicine of São José do Rio Preto, São José do Rio Preto, 15090-000, Brazil; 2 Department Infectious Diseases, Faculty of Medicine of São José do Rio Preto, São José do Rio Preto, 15090-000, Brazil

**Keywords:** COVID-19, asymptomatic testing, prevalence, cancer

## Abstract

**Background:**

The coronavirus disease (COVID-19) pandemic has had enormous consequences in Brazil and worldwide. Patients with cancer affected by COVID-19 are at a higher risk of developing complications and worse outcomes compared to the non-cancer population, particularly the ones on active systemic treatment. Considering the COVID-19’s high transmissibility in asymptomatic and pre-symptomatic patients, we sought to determine the prevalence of COVID-19 infection in patients with solid cancers receiving systemic therapy in a Brazilian public health hospital. Furthermore, we studied whether socio-economic status was associated with prevalence.

**Methods:**

Consecutive asymptomatic patients undergoing treatment for solid tumours at the chemotherapy and infusion centre of Hospital de Base were enrolled. Patients were prospectively tested for severe acute respiratory syndrome coronavirus 2 RNA real-time polymerase chain reaction with nasal and oropharyngeal swabs immediately prior to treatment. A socio-economic survey was carried out prior to testing. Demographic and socio-economic characteristics were summarised in means, medians and proportions.

**Results:**

From 6 to 13 October 2020, 148 asymptomatic patients were identified. Of those, 41 were excluded, leaving 107 eligible patients. The mean age of the population was 58 years (SD ± 12.6); 54% were female and 90% were self-identified as White. The most common cancer sites were gastrointestinal tract (36%) and breast (25%). Most patients had a metastatic disease (59%) and were on anticancer treatment involving chemotherapy (95%). Regarding socio-economic status, 46% of our population had either primary school or illiterate as their highest educational level. In terms of monthly income, 92% had a personal income inferior to U$380 and 88% a household income inferior to U$585. Of the 107 patients tested, only 1 (0.9%) was positive for COVID-19. This is a 48-year-old man living in an urban area, with primary school educational level and a monthly personal income inferior to U$390.

**Conclusion:**

Despite a high prevalence of COVID-19 in Brazil, our cohort demonstrated a low prevalence of COVID-19 (0.9%) amongst asymptomatic patients with cancer. We hypothesise that patients with cancer, independent of their socio-economic status, are aware of the increased risk of developing a severe disease and are adherent to physical distancing, masking and hygiene measures.

## Introduction

Brazil is currently the third most affected country in case numbers and the second in mortality rate during the coronavirus disease (COVID-19) pandemic, with 11,019,344 cases and 265,411 deaths recorded as of 8 March 2021 [1]. These numbers likely reflect a massive underestimate as in general only symptomatic individuals are tested, whereas contacts who are asymptomatic are not routinely tested or traced [2]. While many countries are seeing COVID-19 cases fall using restrictions and vaccines, Brazil’s COVID-19 outbreak is worse than ever. Daily mortality is still climbing, currently at 2,286 deaths per day as of 11 March 2021, with no perspective of changes in the short term. Such impressive numbers significantly impaired Brazilian healthcare services, leading to hospital bed shortages across the country and even shortage of oxygen supply in some cities such as Manaus [3]. Over 70% of the population depend on Brazil’s unified healthcare system (Sistema Unico de Saúde, SUS), which despite its merits, provides suboptimal care to Brazilians in many fronts. For instance, oncology patients are generally medically underserved, with limited oncology healthcare access and poor delivery of costly medications [2, 4].

In this scenario, the Brazilian healthcare workers endure difficulties delivering basic cancer care within SUS. Cancer patients on active systemic treatment are exposed to unavoidable social contacts, such as with hospital staff, contacts with other patients during treatment, commuting to hospital in public transportation etc, and, therefore, are at a higher risk of the COVID-19 contagion compared to a non-cancer population. Furthermore, worse clinical outcomes have been demonstrated in cancer patients with COVID-19, particularly when undergoing chemotherapy [5]. Systematically testing cancer patients for COVID-19 through real-time polymerase chain reaction (RT-PCR) prior to chemotherapy administration has been studied and is recommended by the European Society of Medical Oncology as an attempt to minimise the risk of COVID-19 transmission in healthcare facilities [6, 7]. Nevertheless, testing is costly, particularly in a scarce resource setting such as in low and middle-income countries. In addition, data with regard to the prevalence of COVID-19 amongst asymptomatic patients with cancer is conflicting [8–10]. There is no randomised evidence supporting the benefits or cost-effectiveness of this practice. The pandemic will persist longer than initially thought and a sustainable strategy is urged. The Brazilian current healthcare system cannot afford to test all asymptomatic patients receiving chemotherapy. Thus, these facilities may be of high risk for COVID-19 transmission, as asymptomatic and pre-symptomatic transmissions seem to be responsible for a significant number of cases [11].

Herein we carried out a quality improvement intervention that consisted of routinely testing asymptomatic patients with cancer on active anti-cancer treatment at Hospital de Base (HB) de (São José do Rio Preto, Brazil) for COVID-19 through RT-PCR. We described the prevalence of COVID-19 amongst these patients, as well as correlated the prevalence of COVID-19 with socio-economic and educational status of the investigated population.

## Methods

This is a pilot quality improvement study approved by the ethics committee of the Faculty of Medicine of São José do Rio Preto, São Paulo, Brazil. Informed consent was waived as the proposed intervention is considered the standard of care and not experimental [6, 7].

### Patients

Between 6 and 13 October 2020, asymptomatic patients with solid tumours receiving active cancer treatment at the chemotherapy infusion centre of HB Onco were consecutively enrolled. To be included, patients had to clear a clinical triage and receive anti-cancer treatment via the public healthcare system; therefore, none of the patients included had private health coverage. The clinical triage comprises standard vital signs assessment and anamnesis for COVID-19 symptoms (including fever, defined as body temperature ≥37.8ºC/100.5ºF, cough, headache, loss of taste or smell, shortness of breath and diarrhoea) and/or close contact with COVID-19-infected hosts are carried out.

In our study cohort, asymptomatic patients who passed the clinical screening were prospectively screened for COVID-19 with testing for severe acute respiratory syndrome coronavirus 2 (SARS-CoV-2) RNA in respiratory specimens (nasal and pharyngeal swabs), which were carried out at the Molecular Biology Laboratory at the Faculty of Medicine of São José do Rio Preto, by RT-PCR, immediately prior to systemic treatment. If the test was positive for SARS-CoV-2 RNA, patients and the local healthcare authority were informed. Patients who tested positive were counselled to adhere to social distancing and treatment was delayed for 14 days.

Data were abstracted from electronic patients’ records including demographics characteristics, cancer staging and ongoing treatment. At the time of RT-PCR testing, a routine socio-economic survey was filled, including educational level, place of residence (urban or rural area) and monthly income data. Data tabulation and analysis were carried out using Microsoft Excel (version 16.35, Microsoft).

## Results

Overall, 149 patients received systemic treatment at our centre during the studied period. One patient failed the COVID-19 clinical screening, as he presented with cough and fever, and was referred to the COVID-19 respiratory unit, ultimately testing negative for COVID-19. Of 148 patients, 107 were tested for COVID-19. Forty-one patients were excluded: 15 declined to be tested in the study, 16 had hematological malignancies and 10 were not receiving chemotherapy (only bisphosphonates and/or gonadotropin-releasing hormone agonists). Demographics and socio-economic data are presented in detail in Table 1. Our patients are geographically representative of São José do Rio Preto and surrounding cities (~2 million inhabitants), located in the countryside of the state of São Paulo, Brazil. All patients included were receiving active systemic treatment (Table 1).

One asymptomatic patient (1/107, 0.9%) tested positive for SARS-CoV-2 RNA RT-PCR. He had been hospitalised 6 days prior to COVID-19 testing, receiving antibiotics for an uncomplicated urinary tract infection, and was discharged after 3 days of admission. After the COVID-19 diagnosis, he developed an initially pauci-symptomatic acute respiratory distress syndrome but experienced a complicated clinical course with severe pulmonary embolism in the left lung. He was admitted and underwent intra-arterial pulmonary thrombolytic treatment using alteplase, with clinical improvement and was finally discharged after 10 days of admission due to COVID-19. One hundred and six patients tested negative for SARS-CoV-2 RNA (99.1%) and had no symptoms of COVID-19 until last follow-up.

The socio-economic survey was filled by 102 (95.3%) patients, and it showed that 45.7% had either primary education (*n* = 45) or were illiterate (*n* = 4); 26.1% (*n* = 28) had middle education; 20.5% (*n* = 22) had higher education; and 7.5% (*n* = 8) of our patients had University Education (Table 1). In terms of monthly income, 92.1% (*n* = 94) reported a personal monthly income inferior to US$390; in regards to household incomes, there is a slight increment, with 65.7% (*n* = 67) reporting monthly household incomes inferior to US$390, 22.5% (*n* = 23) between US$390 and US$585, and 11.7% (*n* = 12) between US$585 and US$975. None of our patients had monthly household incomes greater than US$975.

Most patients included were receiving systemic treatment based on chemotherapy either as monotherapy or in combination with other agents (95.3%); other types of treatment included anti-HER2 therapies as maintenance (trastuzumab and/or pertuzumab, 1.8%) and biphosphonates (2.8%). The majority of the patients had metastatic disease (58.9%) and the most frequent site of cancer was the gastrointestinal tract (37.4%).

## Discussion

Our data revealed a low rate of COVID-19 in a cohort of asymptomatic cancer patients undergoing active systemic treatment in Brazil, with 1 patient out of 107 (0.9%) testing positive. Although systematic testing for SARS-CoV-2 PCR-RNA prior to each cycle of systemic therapy in cancer patients has been recommended, our findings suggest that this practice may not be necessary [6]. On the contrary, these resources might be better used to test symptomatic patients or contact tracing.

During the study time, the city of São Jose do Rio Preto was one of the epicentres of COVID-19 in the countryside of the state of São Paulo: COVID-19 incidence reached 150.1 per 100,000 residents, compared to 87.4 in the state of São Paulo and 100.8 in Brazil (numbers referring to symptomatic patients, as there is not a policy for testing asymptomatic persons in Brazil). These crude data show the high rates of transmissibility of COVID-19 in our region, which is discrepant compared to the low rate of COVID-19 in asymptomatic patients with cancer found in our study. The reasons for this finding are unknown.

We speculate that patients with cancer are more adherent to protective measures against the COVID-19 contagion, such as hand washing, social distancing and mask-wearing, as well as avoiding social gatherings, impacting their low infection rate. This conclusion has also been deduced by Shah *et al* [10], who found 0.64% positivity in their New York cohort. In line with this study, Meti *et al* [9] tested a similar cohort in Toronto and found 0% positivity rate of COVID-19 [9]. Both cohorts, however, had no minorities representation and had no data in terms of economic or educational status. Nevertheless, one can suppose that given that the studies were conducted in USA and Canada, their cohorts had higher income and higher educational level compared to ours. Our cohort consisted of Latinos whom in 84% of the cases had a monthly household income inferior to US$585, and whom in 72% of the cases had not received high education, but despite that they had similar results in terms of low COVID-19 positivity (0.9%). We hypothesise that cancer patients regardless of their socio-economic and educational status are aware of their increased risks if infected by SARS-CoV-2 and are more adherent to hygienic measures such as physical distancing, hand washing and masking. Further studies are needed to investigate the behaviour of cancer patients towards COVID-19 preventive measures in comparison to a non-cancer population.

Our quality research approach was limited by the small sample size, no SARS-CoV-2 Immunoglobulin G serological testing and short patient follow-up. However, understanding the value of systematic COVID-19 testing prior to chemotherapy, is a relevant and sensitive matter as we move to the second and subsequent waves of the pandemic, with new virus variants, especially in countries with limited resources, such as Brazil. Our data provide some assurance that with proper patient education, systematic COVID-19 testing prior to chemotherapy may not be substantial to patients undergoing systemic treatment.

## Conclusion

In our cohort, the prevalence of COVID-19 in asymptomatic patients with cancer undergoing active systemic therapy was as low as 0.9%. We hypothesise that patients with cancer are more adherent to the pandemic control measures in comparison to the general population, therefore explaining the low prevalence observed in this study despite a high contagion rate in Sao Jose do Rio Preto at the same period. Our data support omitting routine COVID-19 testing for asymptomatic patients with cancer on systemic therapy, which may save important resources, particularly in low and middle-income settings.

## Funding

This work was supported by FUNFARME – Fundação Faculdade Regional de Medicina de São José do Rio Preto.

## Disclosures

DVA: Received honoraria from MSD, Pfizer and GSK.

AFF: Received honoraria from Astrezeneca.

LAF, BB, MLN and ML have no conflicts to disclose.

## Figures and Tables

**Table 1.** Demographic, cancer-related and socio-economic data of the participants.

Characteristic	*N* (%)
Age (median [min, max])	58 (25–91)
Female	58 (54.2)
Race (patient reported)	
White	96 (89.7)
Brown	6 (5.6)
Black	5 (4.6)
Educational level	
Illiterate	4 (3.7)
Primary	45 (42.0)
Middle	28 (26.1)
High	22 (20.5)
University	8 (7.4)
Place of residence	
Urban	93 (91.1)
Rural	9 (8.9)
Individual income (US$, monthly)	
<190	32 (31.3)
190–380	62 (60.7)
381–580	7 (6.8)
580–965	1 (0.9)
>965	0 (0)
Household income (US$, monthly)	
<190	12 (11.7)
190–380	55 (53.9)
381–580	23 (22.5)
580–965	12 (11.7)
>965	0 (0)
Primary cancer site	
GI tract	39 (36.4)
Breast	27 (25.2)
Lung	13 (12.1)
Head and neck	12 (11.2)
Others	16 (14.9)
Current cancer status	
Non-metastatic	44 (41.1)
Metastatic	63 (58.9)
Treatment type	
Chemotherapy-based	102 (95.3)
Others	5 (4.7)

A total of 107 patients were included. Of the 107, only 102 (95.3%) filled the socio-economic survey.
